# A Monolayer System for the Efficient Generation of Motor Neuron Progenitors and Functional Motor Neurons from Human Pluripotent Stem Cells

**DOI:** 10.3390/cells10051127

**Published:** 2021-05-07

**Authors:** Alessandro Cutarelli, Vladimir A. Martínez-Rojas, Alice Tata, Ingrid Battistella, Daniela Rossi, Daniele Arosio, Carlo Musio, Luciano Conti

**Affiliations:** 1 Laboratory of Stem Cell Biology, Department of Cellular, Computational and Integrative Biology-CIBIO, University of Trento, 38123 Trento, Italy; alessandro.cutarelli@unitn.it (A.C.); alice.tata@unitn.it (A.T.); ingrid.battistella@studenti.unitn.it (I.B.); 2 Institute of Biophysics (IBF), Trento Unit, National Research Council (CNR) & LabSSAH, Bruno Kessler Foundation (FBK), 38123 Trento, Italy; vlalx.mr@gmail.com (V.A.M.-R.); daniele.arosio@cnr.it (D.A.); carlo.musio@cnr.it (C.M.); 3 Laboratory for Research on Neurodegenerative Disorders, Istituti Clinici Scientifici Maugeri IRCCS, 27100 Pavia, Italy; daniela.rossi@icsmaugeri.it

**Keywords:** induced pluripotent stem cells, hiPSC, spinal motor neurons, motor neuron progenitors, cellular models, spinal muscular atrophy

## Abstract

Methods for the conversion of human induced pluripotent stem cells (hiPSCs) into motor neurons (MNs) have opened to the generation of patient-derived in vitro systems that can be exploited for MN disease modelling. However, the lack of simplified and consistent protocols and the fact that hiPSC-derived MNs are often functionally immature yet limit the opportunity to fully take advantage of this technology, especially in research aimed at revealing the disease phenotypes that are manifested in functionally mature cells. In this study, we present a robust, optimized monolayer procedure to rapidly convert hiPSCs into enriched populations of motor neuron progenitor cells (MNPCs) that can be further amplified to produce a large number of cells to cover many experimental needs. These MNPCs can be efficiently differentiated towards mature MNs exhibiting functional electrical and pharmacological neuronal properties. Finally, we report that MN cultures can be long-term maintained, thus offering the opportunity to study degenerative phenomena associated with pathologies involving MNs and their functional, networked activity. These results indicate that our optimized procedure enables the efficient and robust generation of large quantities of MNPCs and functional MNs, providing a valid tool for MNs disease modelling and for drug discovery applications.

## 1. Introduction

Motor neurons (MNs) are cells located in specific areas of the central nervous system (CNS), including the cerebral cortex (upper MNs), brain stem and spinal cord (lower MNs) [1]. They are involved in governing voluntary actions and in general body movements through the participation and contribution of large neuronal circuits. MNs are the only cells in the body that can directly stimulate skeletal muscles, thus modulating their contraction. Their loss results in diseases such as spinal muscular atrophy (SMA) and amyotrophic lateral sclerosis (SLA) [2,3]. At present, pluripotent stem cells (PSCs) represent the most promising tool for the generation of large populations of human neural stem cells, committed progenitors and neurons in vitro, and for the establishment of patient-specific neuronal disease models [4,5,6]. Various methods have been developed for MNs production from PSCs, both of embryonic and somatic origin, generated by means of reprogramming techniques [7,8]. The protocols reported in literature generally are based on a multistep process that includes neural induction, differentiation and maturation phases. The first step can be achieved both by the formation of embryoid bodies (EBs) or in monolayer conditions [9,10,11]. Once the neural precursors are obtained, the following steps involve the patterning into MN progenitor cells (MNPCs) by mirroring in vitro the spinal cord developmental-relevant timing and physiological events, and the subsequent maturation of this cell population into mature MNs [12,13].

Several protocols published during the last years reported a high variability in terms of the efficiency and time required for MNs generation, thus proving the importance for the field to develop a rapid, efficient and robust strategy process of differentiation [12,13,14].

To date, MNs have been successfully differentiated from human embryonic stem cells (hESCs) and human induced PSCs (hiPSCs) by direct differentiation, or from somatic cells (i.e., fibroblasts) by an induction process based on the overexpression of specific transcription factors for the conversion of somatic cells towards MNs. Most of the xeno-free chemically defined available protocols for the generation of hiPSC-derived neurons are based on floating EB formation, a step that makes the process longer and that, due to the complex EB structure, does not allow proper monitoring of the differentiation [15,16,17,18,19,20,21]. To bypass these limitations of the EB-based procedure, some protocols have been developed for differentiating neural progenitor cells (NPCs) in monolayer conditions, yet most of them are based on dual-SMAD inhibition during the early stage of neuralization [22,23,24,25].

In this study, we report a new optimized monolayer procedure for the rapid and efficient differentiation of hiPSCs into mature MNs. The protocol is based on fully defined culture conditions that allow a nearly pure MNPC population to be obtained in 13 days. Moreover, we tested the molecules that allow stable MNPC expansion to rapidly produce large stocks of cryopreservable MNPCs starting from limited amounts of hiPSCs. By applying this protocol, within 30–40 days in vitro (DIV) the MNPC population can mature into functionally active MNs with ~85% of efficiency. The MNs population showed HB9, ISLET-1 and ChAT immunoreactivity, and the expression of their relative transcripts. These hiPSC-derived MNs also exhibited spontaneous firing as assayed by multielectrode arrays (MEAs) and action potential production in response to a current-clamp setting current injection. Also, these MNs exhibited variation in their intracellular Ca^2+^ concentration ([Ca^2+^]i) when exposed to drugs acting on the ion channels, thus further proving their mature functional nature. Finally, this protocol was exploited to differentiate hiPSCs from a SMA patient and a healthy individual control.

On the whole, our study provides an optimized approach to robustly induce a rapid and highly efficient conversion of hiPSCs into enriched populations of MNPCs and fully mature MNs exploitable for biochemical and functional studies, and for the modelling of human CNS pathologies affecting MN populations.

## 2. Materials and Methods

### 2.1. Cell Culture

Human hiPSCs were obtained from ThermoFisher Scientific with verified pluripotency and normal karyotype. GM24474 (https://www.coriell.org/0/Sections/Search/Sample_Detail.aspx?Ref=GM24474&Product=CC) and GM24468 (https://www.coriell.org/0/Sections/Search/Sample_Detail.aspx?Ref=GM24468&Product=CC) fibroblast-derived hiPSC lines were obtained from the Coriell Institute for Medical Research with verified pluripotency and contamination-free. All the hiPSC lines used in this study were cultured in feeder-free conditions in chemically defined Essential 8 (E8) medium on Geltrex-coated tissue culture plastic plates. Medium was changed every 24 h and colonies were passaged by chemical-based procedure with dissociation solution (DPBS without Ca^2+^/Mg^2+^ supplemented with 0.5 mM EDTA). Briefly, 70–80% confluent cultures were washed twice with DPBS W/O Ca^2+^/Mg^2+^, incubated for 3–5 min at 37 °C with dissociation solution and then detached in E8 medium, collected and spun at 200 g for 5 min. Pellet was resuspended in E8 medium and 1–1.5 × 10^4^ cells/cm^2^ were plated on Geltrex-coated tissue culture plastic. Cultures were maintained at 37 °C, 5% CO_2_. All cell culture reagents were purchased from ThermoFisher Scientific (Monza, Italy).

### 2.2. MNPCs and MNs Differentiation

For the differentiation process, 70% confluent hiPSC cultures were dissociated as described above and then plated on vitronectin-coated plastic in neural induction medium (NIM) composed of Essential 6 (E6) medium (ThermoFisher Scientific) supplemented with 1% GlutaMAX (ThermoFisher Scientific), 10 μM Y-27632 (Santa Cruz Biotechnology, Heidelberg, Germany) and maintained at 37 °C for 48 h. After 48 h, the medium was replaced with NIM without Y-27632; medium was entirely renewed every other day. At day 6, cultures were shifted to E6 medium supplemented with 100 nM all-trans retinoic acid (RA; Focus Biomolecules, Plymouth Meeting, PA, USA) to induce the caudalization of the neuroepithelial cells. Cells were maintained under these conditions for two days and then dissociated and replated on vitronectin-coated plastic in MNP patterning medium (MNP-PM) composed of E6 medium supplemented with 2% B27 supplement (ThermoFisher Scientific), 100 nM RA, 2 μM purmorphamine (PMN; Santa Cruz Biotechnology, Heidelberg, Germany), and 10 μM Y-27632 for the selection of MNPCs. Cells were maintained in these conditions for two days and then medium was replaced with MNP-PM without Y-27632; medium was then renewed every other day.

Once confluent, MNP cultures can be passaged for further expansion by dissociation with StemPro Accutase (ThermoFisher Scientific) and replated on vitronectin-coated plastic in MNPCs expansion medium (MNP-EM) consisting of MNP-PM supplemented with 0.5 mM valproic acid (VPA; Santa Cruz Biotechnology), 3 μM CHIR99021 (Sigma Aldrich, Milano, Italy) and 10 μM Y-27632. Medium was changed two days after plating with MNP-EM without Y-27632 and renewed every other day. Cells can be expanded to confluency for at least 3 passages; cultures can be cryopreserved at this stage.

To induce MNs maturation, MNPCs were dissociated with StemPro Accutase and 2–4 × 10^4^ cells/cm^2^ seeded on vitronectin-coated plastic in MN maturation medium (MN-MM) composed of neurobasal medium (ThermoFisher Scientific) supplemented with 1% GlutaMAX, 2% B27, 100 nM RA, 1.5 μM PMN, 1% N2 supplement (ThermoFisher Scientific), 0.2 μM cAMP (Santa Cruz Biotechnology), 10 ng/mL BDNF (Peprotech), 10 ng/mL GDNF (Peprotech), 10 ng/mL IGF (Peprotech, London UK) and 10 μM Y-27632. After 2 days, the medium was completely renewed with MN-MM without Y-27632 and then partially (50%) renewed twice a week. MNs in these conditions can be long-term cultured to induce full functional maturation.

### 2.3. Immunofluorescence Assay

Cells were washed once with cold DPBS with Ca^2+^/Mg^2+^, fixed with 4% PFA for 15 min at room temperature (RT), permeabilized with permeabilizing solution (0.5% Triton X-100 in PBS) for 15 min at RT and blocked 1 h at RT with blocking solution (5% FBS, 0.3% Triton X-100 in DPBS). Cells were incubated at 4 °C overnight with antibody solution (2% FBS, 0.2% Triton X-100 in PBS) containing the specific primary antibodies, rinsed twice with PBS and incubated with relative secondary antibodies for 2 h at RT. Primary antibodies used in this study included polyclonal anti-aPKC (1:100; Santa Cruz Biotechnology), polyclonal anti-Zo-1 (1:300; ThermoFisher Scientific), monoclonal anti-ISLET1 (1:300; Santa Cruz Biotechnology), polyclonal anti-ISLET1 (1:300; AbCam), polyclonal anti-OLIG2 (1:300; Millipore), monoclonal anti-HB9 (1:100; Santa Cruz Biotechnology), monoclonal anti-TuJ1 (1:1000; Santa Cruz Biotechnology), polyclonal anti-MAP2 (1:100; Santa Cruz Biotechnology), monoclonal anti-NKX2.2 (1:300; Santa Cruz Biotechnology), monoclonal anti-ChAT (1:200, Santa Cruz Biotechnology), monoclonal anti-NESTIN (1:500; RD System), and monoclonal anti-phospho-histone H3 (1:500; Millipore). Secondary antibodies used in this study were Alexa Fluor 488 and 568 (1:500; ThermoFisher Scientific). Nuclei were counterstained with Hoechst 33342 (ThermoFisher Scientific). Negative controls were obtained by omitting the primary antibody. Images were visualized on Leica DM IL LED fluo microscope equipped with 5×/10×/20×/40× objectives and images acquired with Leica DFC450 C camera. For the evaluation of the number of immunofluorescence-labelled cells, 5–8 photographic fields for each sample (at least 2000 cells per each sample were considered; each sample was produced in triplicate) were analyzed and quantified by using Image J software (NIH) or by manual counting.

### 2.4. Gene Expression Analysis

Total RNA was isolated using TriFast (Euroclone) and subsequently used to synthesize cDNA by iScript kit (Bio-Rad). qPCR was then performed using the reaction mix containing SsoAdvanced SYBR green (Bio-Rad), primers (200 nM), cDNA (2.5 ng/μL) and nuclease-free H_2_O. Amplification reaction was performed on RealTime System CFX96TM (Bio-Rad) using the following program: 95 °C for 15 min and then 40 cycles (15 s at 91 °C, 30 s at 60 °C and 31 s at 65 °C) followed by 60 cycles of 5 s from 65 °C to 95 °C increasing the temperature by 0.5 °C per cycle. Quantitative levels for all genes were normalized to housekeeping genes (GAPDH or beta-actin). For MNPC and MN genes, levels were expressed as relative to hiPSCs. Primer sequences used are as follow: Beta-Actin_Fw: 5′GACAGGATGCAGAAGGAGATTACTG, Beta-Actin_Rev: CTCAGGAGGAGCAATGATCTTGAT; hGapdh_Fw: CCACTCCTCCACCTTTGAC, hGapdh_Rev: ACCCTGTTGCTGTAGCCA; hChAT_Fw: CCCTGATGCCTTCATCCA, hChAT_Rev: GTAGGTGGGCACCAGTCTTC; hOlig2_Fw: AGCTCCTCAAATCGCATCC, hOlig2_Rev: ATAGTCGTCGCAGCTTTCG; hHb9_Fw: TGCCTAAGATGCCCGACTT, hHb9_Rev: AGCTGCTGGCTGGTGAAG; hIslet1_Fw: CGTGCCCGCTCCAAGGTGTATCA, hIslet1_Rev: CATTGGGCTGCTGCTGCTGGAGTT; hNestin_Fw: GGAGAAGGACCAAGAACTG, hNestin_Rev: ACCTCCTCTGTGGCATTC-3′; hβ3-tubulin_Fw: TCAGCGTCTACTACAACGAGGC, hβ3-tubulin_Rev: GCCTGAAGAGATGTCCAAAGGC.

### 2.5. Patch-Clamp Electrophysiology

MNPCs differentiated by mean of the protocol described above were dissociated and 2.5 × 10^5^ cells were replated in maturation medium on vitronectin-coated 12-mm plastic coverslips (SPL) and allowed to mature for 30 or 60 days. Patch-clamp recordings were obtained from a total number of 40 hiPSC-derived motor neurons in the whole-cell configuration [26,27]. Pipettes were made on a temperature-controlled PIP6 puller (HEKA) from borosilicate glass capillaries (Harvard Apparatus pipette electrodes had resistance ranging 4–6 MΩ when filled with an intracellular solution containing the following: in (mM) 130 K-gluconate, 10 KCl, 0.1 CaCl_2_, 2.0 MgCl_2_, 1.1 EGTA, 10 HEPES, 2.0 Mg-ATP, and 0.2 Na-GTP, pH 7.3 (280 mOsm). The standard bath solution had the following composition: (mM) 140 NaCl, 4 KCl, 10 HEPES, 2.0 MgCl_2_, 2.0 CaCl_2_ and 10 glucose, pH 7.4 and 290 mOsm. The bioelectrical signals were obtained using an ELC-03XS amplifier (Npi), filtered at 2 kHz with a low-pass filter, and sampled at 10 kHz with an INT-20X interface (Npi). Data were acquired with WinWCP software (©John Dempster, University of Strathclyde, Glasgow, UK). Clampfit (Molecular Devices) and Prism 8 (GraphPad Software, Inc., San Diego, CA, USA) were furtherly employed for data analysis and plotting.

Resting membrane potential (RMP) was measured in bridge balance configuration after gigaseal breaking in. Voltage-dependent conductances were evoked by depolarizing pulses (10-mV steps, −80 to 40 mV, 100 ms) from a holding potential of −70 mV. Then, in current-clamp mode, a series of current pulses required to elicit action potential (AP) were somatically injected. All APs were evoked from an adjusted membrane potential of −50 mV. Single APs were elicited by a 10-ms suprathreshold step and waveform characteristics were determined by differentiating the spike voltage with respect to time (dV/dt). Phase plots were constructed by plotting the first derivative in mV/ms against the membrane voltage. The membrane passive properties were estimated using hyperpolarizing current pulses from 0 to −150 pA in –30 pA steps. Recording quality was monitored on-line incorporating the following criteria: stable RMP, access resistance (<20 MΩ, <20% drift), and holding currents < 100 pA.

Data are presented as mean ± SEM. Data and statistical analyses were performed using WinWCP and OriginPro 9.5 (OriginLab Corporation) Student’s unpaired *t*-test. A value of *p* ˂ 0.05 was considered statistically significant.

### 2.6. Live Calcium Imaging

MNPCs were dissociated and 2.5 × 10^4^ cells/cm^2^ were seeded in a 24-well plate in neuronal maturation medium and allowed to mature for 30 or 60 days. The [Ca^2+^]i variation was evaluated by means of optical fluorimetric recordings with Fura-2AM fluorescent probe [28]. The ratio between the values of light intensity at 340 and 380 nm stimulation was recorded every 3 s. For spontaneous calcium firings, ratio values were recorded every 60 ms. Fura-2AM stock solutions were obtained by adding 50 μg of Fura-2AM to 50 μL of DMSO. Cells were bathed for 45–60 min at 37 °C with 2.5 μL of stock solution diluted in 0.5 mL of conditioned maturation medium for a final Fura-2AM concentration of 5 μM. The medium was then removed and washed 3 times with the extracellular solution used for recordings (in mM: 125 NaCl, 1 KCl, 5 CaCl_2_, 1 MgCl_2_, 8 glucose, and 20 HEPES, pH 7.35). An iMIC (Till Photonics) equipped with polychrome II and proprietary software (version 2.7.0.16) for acquisition and offline analysis was used to measure fluorescence changes. Emitted light was captured by a CCD camera Retiga2000-DC (QImaging) and the objective used was either UCPLFLN 20× NA 0.7 or UPLFLN 10× NA 0.3 (Olympus). Video resolution in Movie S1 was 1600 × 1200 reproduced at 25 fps, 10× objective. Data are expressed as mean measurements ± SEM and *n* represents the number of recorded cells.

### 2.7. High-Density (HD)-MEAs Recordings

MNPCs were plated on the CMOS (complementary metal-oxide-semiconductor)-based HD-MEA, BioChip HD-MEA Stimulo, from 3Brain AG, which consists of 4096 recording channels (in a 64 × 64 grid; 21 × 21 μm^2^, 81 μm pitch) and 16 stimulating channels (in 4 × 4 grid; 21 × 21 μm^2^, 1246 μm pitch). BioChips were sterilized in 70% ethanol for 20 min and rinsed thoroughly with sterilized distilled water (SDW), then left to air dry under a laminar hood. BioChips were then pre-conditioned with neuronal maturation medium overnight at 37 °C and then pre-coated with poly-d-lysine (50 μg/mL) overnight at 37 °C, rinsed thoroughly with SDW and then coated 1 h with vitronectin at 37 °C. After the cells were dissociated, the concentration was diluted to 1000 cells/μL and 85,000 cells were seeded on the HD-MEA chip with an 85 μL droplet. Cells were left to settle for 3–4 h before adding 1.5 mL of neuronal maturation medium. The culture was incubated at 37 °C, 5% CO_2_ and half of the medium was changed every 3–4 days. Multiple recording sessions of 5 min were performed at day 60 of neuronal maturation with a HD-CMOS technology microelectrode array of 4096 microelectrodes (BioCam X, 3Brain) sampled at 17.8 KHz/electrode and analyzed with the integrated brainwave software application.

### 2.8. Statistical Analyses

Values are expressed as the mean ± SD of duplicate cultures and are representative of at least 3 independent experiments. Statistical analyses of data were performed using GraphPad Prism software. The statistical comparison of the various groups was performed with a simple variance analysis (ANOVA), followed by correction for multiple LSD tests (least significant difference). For independent samples, the comparative statistical analysis was carried out with a Student’s *t*-test by applying the Bonferroni post-hoc test. The values were considered statistically significant for *p* < 0.05 (*), *p* < 0.01 (**), *p* < 0.001 (***) and *p* < 0.0001 (****).

## 3. Results & Discussion

### 3.1. Specification of Motor Neurons Progenitor Cells from hiPSCs

**Figure 1:**
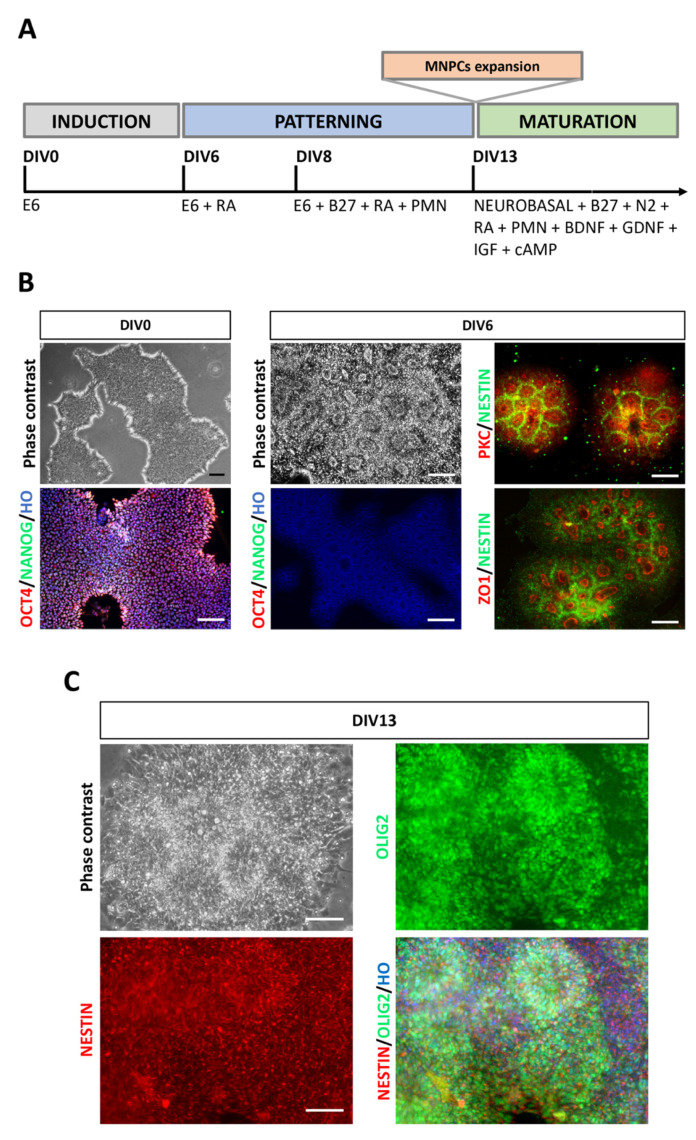
hiPSCs induction into MNPCs in monolayer conditions. (**A**) Schematic representation of MNPCs and MNs induction with all media and factors. (**B**) Representative pictures of the starting (DIV0) hiPSC cultures. At DIV6, cultures exhibit the formation of neural rosettes while showing lack of immunoreactivity for OCT4 and NANOG. These cultures instead show NESTIN immunoreactive signal together with PKC and ZO1 immunolabelling. Nuclei are stained with Hoechst (HO). (**C**) Contrast phase and immunofluorescence pictures of DIV13 cultures showing the presence of MNPCs immunoreactive for NESTIN and OLIG2; most of the cells are double positive for both markers. Nuclei are stained with Hoechst (HO). Scale bars: 100 µm.

To establish a robust and simplified monolayer procedure for rapid and efficient MNPCs from hiPSCs, we focused on mimicking the developmental features underlying spinal cord formation by using the minimum required molecules in a xeno-free environment. Based on the use of E6 medium, and of vitronectin as a coating agent, we optimized the entire monolayer processes for neuralization and spinal cord patterning, which does not require the addition of dual-SMAD inhibitors or EBs formation at any stage (Figure 1A). In these conditions, hiPSCs were consistently differentiated into neuroepithelial cells in 6 days (Figure 1B). At DIV6, the cultures were characterized by the absence of residual hiPSCs as shown by the lack of OCT4^+ve^ and NANOG^+ve^ cells, and by the presence of neural cells arranged in neural rosette structures and exhibiting marked immunoreactivity for NESTIN and for the neural rosette markers ZO1 and aPKC (Figure 1B) [29,30].

At this stage, in order to optimize the patterning of neuroepithelial cells into MNPCs, we tested different timeframes for the exposure to patterning cues, i.e., RA and PMN (a Shh agonist), both recognized molecules known to allow efficient spinal cord specification [31]. Notably, RA has been proved to alter cell morphology and gene expression on neural rosette-stage cultures and, depending on the timing of exposure, it heavily affects MNs generation efficiency, which results negatively if RA is not dispensed within the correct timeframe [13,32].

Specifically, we tested (*i*) condition #1, which included the exposure of DIV6 cultures to RA followed by DIV8 with PMN addition (Figure 1A), and (*ii*) condition #2 in which RA and PMN exposure were anticipated at DIV5 and DIV7, respectively (Figure S1). After RA/PMN exposure, the DIV8 cultures were replated on vitronectin-coated plastic and maintained in patterning conditions until DIV11.

To assess the patterning efficiency, the DIV11 cultures were analyzed by immunofluorescence assay for the expression of OLIG2, a transcription factor expressed by MNPCs. Condition #1 led to significantly higher efficiency at DIV11 with respect to condition #2 (Figure S2, left panel). This result was confirmed also by the qPCR assay (not shown). In the optimized protocol (Figure 1A), the patterning conditions were maintained for an additional two days until DIV13, when nearly all the cells in the culture co-expressed NESTIN and OLIG2 (percentage of NESTIN^+ve^ cells over total number of nuclei: 94.39 ± 4%; percentage of OLIG2^+ve^ cells over total number of nuclei: 85.77 ± 5%; percentage of NESTIN^+ve^/OLIG2^+ve^ cells over total number of nuclei: 85.77 ± 5%; Figure 1C), indicating an efficient specification towards MNPCs.

During neural development, a pool of progenitors co-expressing OLIG2 and NKX2.2 eventually segregates to generate OLIG2^+ve^ MNPCs committed to MNs and NKX2.2^+ve^ progenitors that give rise to spinal interneurons [33]. Therefore, we sought to investigate whether (*i*) this developmental process might be recapitulated in our cultures and (*ii*) if the two above tested patterning conditions could lead to discordant behavior. To evaluate this occurrence, we assessed the percentage of OLIG2^+ve^ and OLIG2/NKX2.2 double-positive cells in DIV11 (early MNPCs) and DIV15 (late MNPCs) cultures. The DIV11 cultures exposed to condition #1 exhibited a significantly higher number of OLIG2^+ve^ cells compared to condition #2 (88 ± 3% and 74 ± 5%, respectively; Figure S2, left panel). Notably, at DIV15 the total number of OLIG2^+ve^ cells decreased in both the conditions, although condition #1 was proven to be more efficient in preserving the number of OLIG2^+ve^ cells (condition #1: 76 ± 3%; condition #2: 50 ± 4%) and thus MNPCs identity in cultures (Figure S2A). Also, we found that condition #1 exhibited a higher efficiency in generating double-positive cells at DIV11 coupled with a 7-fold reduction in OLIG2/NKX2.2 double-positive cells at DIV15 compared to condition #2, suggesting that the former is more efficient in allowing MNPCs generation and their subsequent segregation into OLIG2^+ve^/NKX2.2^−ve^ or OLIG2^−ve^/NKX2.2^+ve^ MN-restricted progenitors (Figure S2B,C). Based on these results, patterning condition #1 was selected as the choice for the next experiments.

### 3.2. Stable Short-Term Expansion of hiPSC-Derived MNPCs

**Figure 2:**
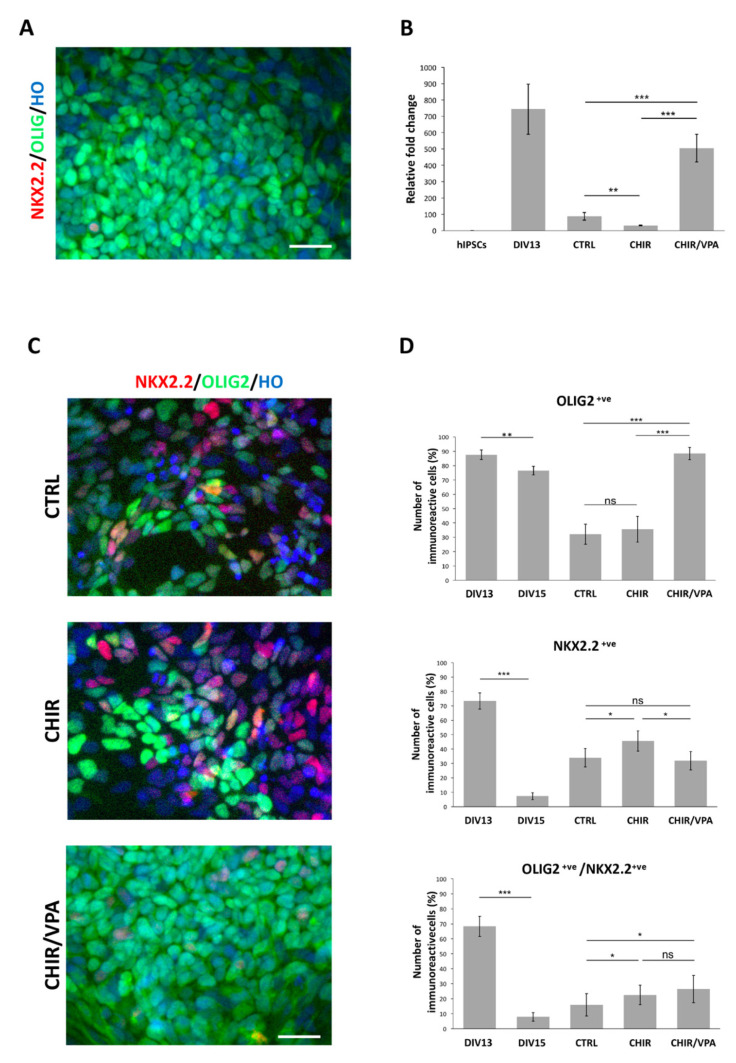
CHIR and VPA treatment allows efficient MNPCs expansion. (**A**) DIV15 MNPCs immunoreactivity for OLIG2 and NKX2.2. Nuclei are stained with Hoechst (HO). (**B**) qPCR analysis for OLIG2 transcript levels in hiPSCs, DIV13 MNPCs and cultures expanded for three passages in CTRL, CHIR and CHIR/VPA conditions. (**C**) Representative pictures showing OLIG2 and NKX2.2 immunoreactivity on cultures expanded for three passages in CTRL, CHIR and CHIR/VPA conditions. Nuclei are stained with Hoechst (HO). (**D**) Graphs reporting the number of OLIG2, NKX2.2 and OLIG2/NKX2.2 double-positive cells (top, central and bottom panel, respectively) in DIV13 and DIV15 MNPCs and cultures expanded for three passages in CTRL, CHIR and CHIR/VPA conditions. The percentage is normalized over the total number of cells assessed by HO staining. Scale bars: 15 µm. Data are expressed as the means ± STDV (*n* = 3 biologically independent experiments). *p* < 0.05 (*), *p* < 0.01 (**), and *p* < 0.001 (***) not significant (ns) according to ANOVA analysis.

To obtain enough material for biochemical investigations it would be fundamental to expand MNPCs while preserving their ability to produce MNs. Since condition #1 proved to be highly efficient in specifying MNPCs, we tested whether it was possible to expand the DIV13 cultures for some passages without affecting the fraction of OLIG2^+ve^ MNPCs. We first examined if passaging the cells once in the standard conditions used for their MNPCs patterning would consent such a step of expansion. We found that two days after the first passage, OLIG2^+ve^/NKX2.2^−ve^ cells were prevailing in culture while very few OLIG2^+ve^/NKX2.2^+ve^ and OLIG2^-ve^/NKX2.2^+ve^ cells were detectable (Figure 2A), thus confirming the above-mentioned segregation process. However, after three passages, the fraction of OLIG2^+ve^ MNPCs in culture was decreased to 31 ± 5%, while the percentages of OLIG2^+ve^/NKX2.2^+ve^ and OLIG2^-ve^/NKX2.2^+ve^ increased (14 ± 7% and 17 ± 6%, respectively). Also, 36 ± 4% of the cells in culture were OLIG2^-ve^/NKX2.2^−ve^ due to the occurrence of spontaneous differentiation (not shown). These results indicated that the patterning culture conditions were poorly amenable for MNPCs expansion, similarly to what was already described in a previous report [34]. The addition of RA, PMN, CHIR99021 (a WNT agonist), SB431542 (an inhibitor of Activin/Nodal signaling), DMH1 (an inhibitor of BMP signaling) and valproic Acid (VPA, a histone deacetylase inhibitor) has been reported to sustain stable MNPCs expansion in culture for 3–5 passages [34]. We thus tested some of these molecules in our culture conditions by expanding the DIV13 MNPCs cultures for three passages in CTRL conditions (composition: standard patterning medium), CHIR conditions (composition: standard patterning medium supplemented with CHIR99021) and CHIR/VPA conditions (composition: standard patterning medium supplemented with CHIR99021 and VPA). We then tested the efficacy of these conditions in maintaining proliferation capacity while preserving MNPCs identity. The analysis of the dividing cells in culture, by the examination of pHH3 immunoreactive cells, showed that the CHIR cultures underwent a significant reduction in proliferation compared to both other conditions, whereas the CHIR/VPA condition did not show any significant difference compared to the CTRL condition (Figure S3). Moreover, qPCR analysis indicated that the CHIR/VPA cultures exhibited a higher performance in preserving elevated OLIG2 transcript levels when compared to the unpassaged DIV13 cultures and to the CTRL and CHIR conditions (Figure 2B). Finally, the analysis of the immunoreactive cells for OLIG2 and NKX2.2 confirmed the CHIR/VPA condition as the most efficient in maintaining OLIG2^+ve^ cells compared to the CTRL and CHIR conditions (Figure 2C; Figure 2D, top panel). The number of NKX2.2^+ve^ cells resulted to be higher in the CHIR condition compared to the others, whereas no significant difference was observed between the CTRL and CHIR/VPA conditions (Figure 2D, central panel). Notably, the fraction of OLIG2^+ve^/NKX2.2^+ve^ cells was significantly higher in the CHIR and CHIR/VPA conditions with respect to the CTRL (16 ± 8%, 22 ± 7% and 26 ± 8% in CTRL, CHIR and CHIR/VPA conditions, respectively; Figure 2D, bottom panel), thus possibly indicating that the CHIR99021 treatment partially preserves the maintenance of more plastic OLIG2 and NKX2.2 double-positive MNPCs.

These results identified the CHIR/VPA condition as the most competent in sustaining proliferative MNPCs state while preserving their MNPCs identity for at least three passages. Notably, compared to the MNPCs expansion conditions previously reported, the CHIR/VPA expansion medium did not include SB431542 and DMH inhibitors, thus consenting to further reduce the minimum required factors in the culture system. Our results indicate that RA and PMN are required to specify MNPCs whereas CHIR and VPA resulted mandatory for proliferation and cell identity maintenance during cell passaging. More specifically, we found that CHIR treatment disturbed the MNPCs proliferation and produced significant negative effects on OLIG2 transcript level expression. On the other side, VPA treatment had no effects neither on OLIG2 transcript levels nor on cell proliferation (not shown). We can thus speculate that CHIR and VPA act in synergy to preserve the MNPCs identity during proliferation with VPA, possibly responsible for neurogenesis repression by the activation of Notch signaling and CHIR contributing to maintain the MNPCs identity [35,36,37].

We found that after the third passage, the cultures underwent a progressive loss in expansion efficiency, indicating that further refinements are required for achieving a longer-term expansion. Nevertheless, the expansion for three passages permits to obtain about 2 × 10^7^ MNPCs starting from 3 × 10^5^ hiPSCs (not shown), thus producing large batches that can be directly used for experimental needs or frozen and thawed with over 90% recovery of viable cells.

### 3.3. Efficient Differentiation of hiPSC-Derived MNPCs into MNs

**Figure 3:**
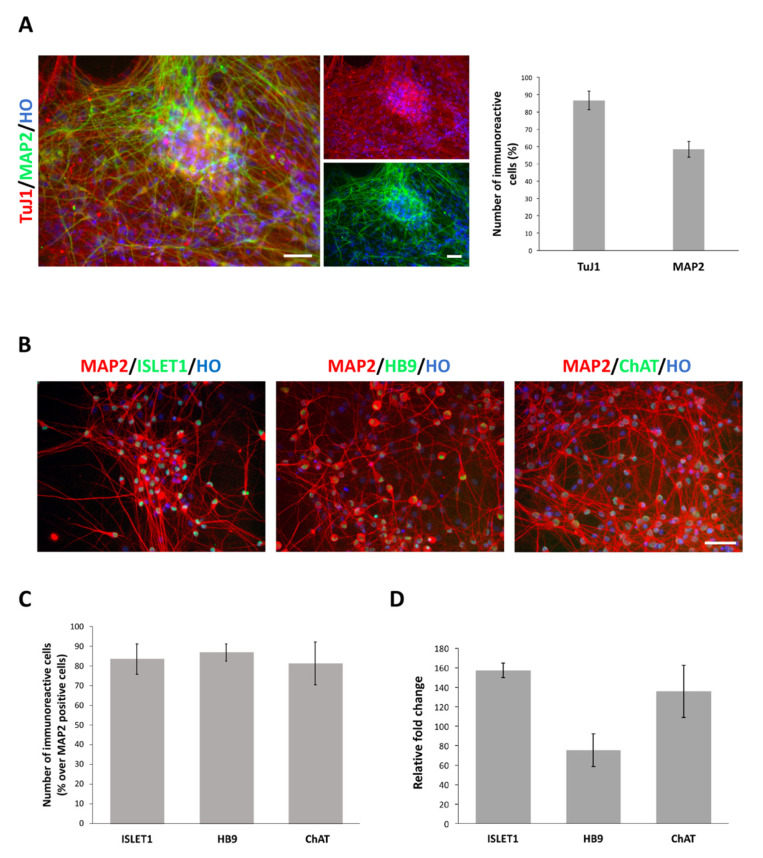
MNPCs efficiently differentiate into MNs when exposed to 4 weeks of neuronal maturation conditions. (**A**) Representative pictures of 4-weeks cultures showing that the population is nearly homogeneously composed by neuronal cells immunoreactive for Tuj1 (top right panel), MAP2 (bottom right panel) along with merged picture (left panel). Nuclei are stained with Hoechst (HO). Scale bar: 100 µm. The graph reports the quantification of TuJ1 and MAP2 immunoreactive cells in culture over the total number of cells assessed by HO staining. (**B**) Representative pictures of ISLET1, HB9 and ChAT immunoreactive MNs. Nuclei are stained with Hoechst (HO). Scale bar: 50 µm. (**C**) Graphs showing number of ISLET1-, HB9- and ChAT-positive cells over the total number of MAP2 immunoreactive cells (lower panel). (**D**) qPCR analysis for ISLET1, HB9 and ChAT transcript levels. Data are expressed as the means ± STDV (*n* = 3 biologically independent experiments).

Cultures after the patterning stage, or expanded MNPCs, are amenable to undergo MNs specification by exposure to defined culture conditions that allow neuronal maturation (Figure 1A). qPCR assay for nestin and β3-tubulin transcripts performed on samples at different stages along the whole process of differentiation, showed that the nestin transcript levels decreased over time, with β3-tubulin exhibiting an opposite pattern of expression (Figure S4). After 4 weeks of exposure to neuronal maturation conditions, ~85% of the cells in culture exhibited TuJ1 immunoreactivity, an early-stage neuronal marker, thus indicating that they underwent efficient neuronal commitment (Figure 3A). Also, nearly 60% of these cells co-expressed MAP2^+ve^, a late-stage neuronal marker (Figure 3A). Immunofluorescence assays for the MNs markers ISLET1, HB9 and ChAT (choline acetyl transferase) showed that, at this stage, ~80–85% of the MAP2^+ve^ cells co-expressed these proteins (Figure 3B,C). The MN identity of the cultured neurons was further confirmed by qPCR assay showing that the transcripts for these markers were all highly induced after 4 weeks of exposure to neuronal maturation conditions (Figure 3D). The cultures maintained in pattern condition #1 exhibited a superior efficiency also in terms of the fraction of TuJ1 and MAP2 immunoreactive cells (Figure S5A), as well as in terms of the expression levels of the transcripts for the MNs markers (Figure S5B).

**Figure 4:**
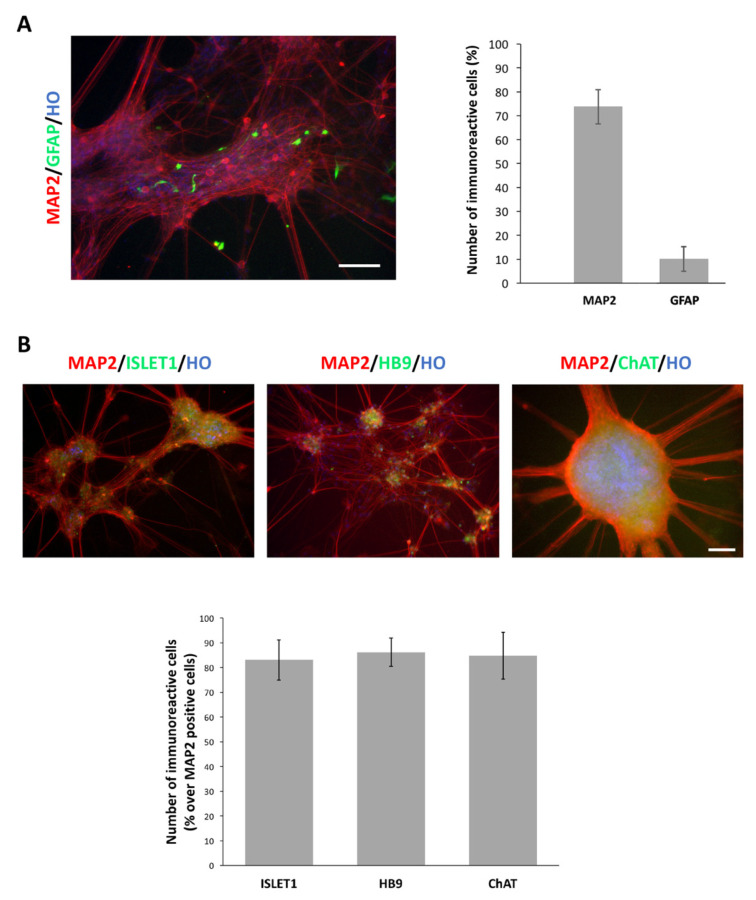
MNs can be long-term maintained in culture. (**A**) Representative picture of MAP2 and GFAP immunoreactive cells in 8-weeks neuronal mature cultures (left panel). Nuclei are stained with Hoechst (HO). Graph reporting the numbers of immunoreactive cells for MAP2 and GFAP at this stage over the total number of cells assessed by HO staining (right panel). (**B**) Representative pictures of MAP2^+ve^ MNs that co-express ISLET1, HB9 and ChAT (upper panel). Nuclei are stained with Hoechst (HO). Graph showing the number of cells positive for MNs markers over the total number of MAP2^+ve^ cells (lower panel). Scale bar: 100 µm. Data are expressed as the means ± STDV (*n* = 3 biologically independent experiments).

Finally, we tested if longer maintenance in culture led to the homogeneous late maturation of the neurons in culture (i.e., increased fraction of MAP2^+ve^ cells). After 8 weeks of exposure to neuronal maturation conditions, the fraction of MAP2^+ve^ cells exhibited a 25% increase (75% of the total number of cells in culture). Also, at this stage we found ~10% of GFAP^+ve^ cells in culture (Figure 4A), indicating that the protocol efficiently favored neuronal commitment and strongly reduced astroglial differentiation. The cultures were also stained for MNs markers, revealing a high percentage (~80–85%) of MAP2^+ve^ cells co-expressing ISLET1, HB9 and ChAT (Figure 4B).

### 3.4. MNPCs Differentiate into Functional MNs

**Figure 5:**
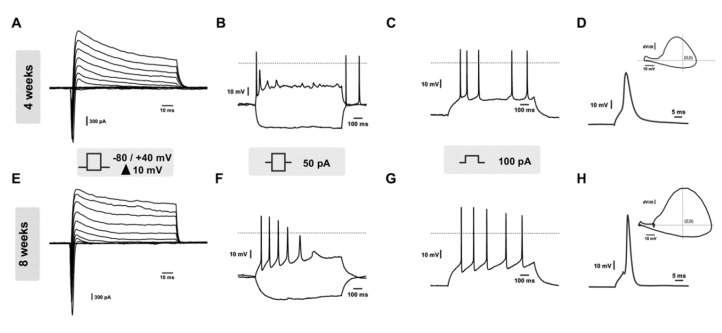
Functional electrical activity of hiPSC-derived MNs. (**A**,**E**) Representative voltage-gated ion current traces elicited during the stimulation through 10 mV voltage steps as shown in the stimulation protocol (middle box). (**B**,**F**) Active membrane responses to hyperpolarizing (−50 pA) and depolarizing (50 pA) current injection (1 s). (**C**,**G**) Examples of firing characteristics in 4- and 8-weeks mature cultures as response to 100 pA/1 s current injections. (**D**,**H**) Representative recordings of single AP elicited upon brief (10 ms) current pulse. Inset: phase plots (dV/dt versus membrane voltage) constructed from example records. For the whole figure, the upper and lower panels represent 4-weeks and 8-weeks of maturation, respectively.

To determine the functional properties of the cells at 4 and 8 weeks of neuronal maturation, we analyzed their effective electrical and biophysical properties by whole-cell patch-clamp. In the voltage-clamp configuration, the voltage step stimulation elicited sharp Na^+^-associated inward and K^+^-associated outward current components in both the time points (Figure 5A,E). Notably, the 8-week mature cells showed superior amplitude of the currents measured at −20 mV for the inward currents and +40 mV for the outward currents compared to the 4-weeks mature cells, thus indicating increased neuronal functional activity over time (Figure S6A).

The neuronal membrane properties were also analyzed by injecting hyperpolarizing and depolarizing current pulses (−50 and 50 pA, respectively), leading to clear dynamic membrane responses and action potentials (APs) in both the time points (Figure 5B,F), whereas the stronger depolarization currents (100 pA) triggered an increased number of evoked APs (Figure 5C,G). The single AP analysis shows different kinetics between the two maturation stages in the corresponding phase plot (Figure 5D,H insets) and the passive membrane properties did not change over time (Figure S6B). The intrinsic excitability analysis displayed a statistically significant increase in AP amplitude in the 8-weeks mature MNs compared to the 4-weeks cultures. However, no change in rheobase, AP half-width, or threshold were observed (Figure S6C). Taken together these data demonstrated that MNs show a fully identifiable neuronal activity at both time points, and the main functional differences between these maturation stages were in the ionic currents and APs amplitude. Accordingly, the reduced amplitude of the inward current might represent the electrical correlate underlying the smaller AP amplitude of the 4-weeks compared to the 8-weeks maturation period.

**Figure 6:**
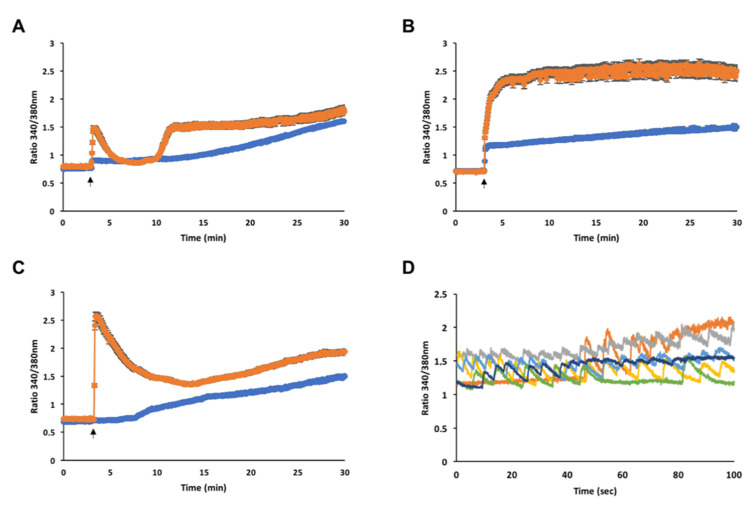
Glutamate receptors activity increases during neuronal maturation. (**A**) Bath application of 100 µM NMDA, (**B**) AMPA and (**C**) kainate, on 4-weeks (blue line) and 8-weeks (orange line) mature MNs. Black arrows indicate agonists application. NMDA 4-weeks (*n* = 80), 8-weeks (*n* = 58); AMPA 4-weeks (*n* = 90), 8-weeks (*n* = 120); kainate 4-weeks (*n* = 56), 8-weeks (*n* = 50). (**D**) Spontaneous calcium firings of 6 representative cells in 8-weeks mature cultures.

To further assess the MNs functional activity we evaluated single cell changes in [Ca^2+^]i, following bath application of glutamate receptor agonists on the 4-weeks and 8-weeks mature cultures. The NMDA treatment triggered a fast increase in [Ca^2+^]i at both maturation time points. Notably, the higher ratio (1.48 ± 0.04) of the peak rapidly achieved following stimulation and the biphasic response (ratio at 30 min after treatment: 1.80 ± 0.06) in the 8-weeks mature MNs was suggestive of increased receptor functional activity, and more neuronal mature identity, compared to the 4-weeks mature MNs. Indeed, the latter exhibited only a minor peak with a ratio that progressively increased up to 1.60 ± 0.02 by the end of the 30-min observation (Figure 6A). AMPA application led to a fast and persistent increase in [Ca^2+^]i in the MNs at both the time points. Furthermore, the 8-weeks mature MNs showed higher ratiometric values up to 2.61 ± 0.08 along with a scattered profile following stimulation, once again indicating increased receptor functional activity at this stage compared to the 4-weeks mature MNs, which slowly increased the ratio until the end of the observation, up to 1.49 ± 0.03 (Figure 6B). Kainate stimulation on the 8-weeks mature MNs elicited a fast [Ca^2+^]i increase, for a ratio up to 2.57 ± 0.07, followed by a biphasic response leading the ratio increasing by 1.93 ± 0.04 at the end of the experiment. Only a delayed [Ca^2+^]i increase slowly reaching a ratio up to 1.49 ± 0.01 was observed in the 4-weeks mature MNs following stimulation (Figure 6C; Movie S1), indicating also for the kainate receptors, increased functional activity over time.

These data revealed an increased response of hiPSC-derived MNs to the excitatory neurotransmitter glutamate over time. The observed increase in the receptors activity may be due to a variated number of exposed surface ligand-gated ion channels, or may be caused by a different combination of subunits in the receptor composition that occurs over neuronal maturation. Further studies are needed to corroborate these hypotheses. However, the presence of mature and active MNs was also confirmed by detecting spontaneous calcium waves in ~10% of recorded cells (Figure 6D). Interestingly, the basal ratio value of resting, unstimulated cells is about 0.7 (Figure 6A–C), whereas the ratio of spontaneously firing cells always waved above 1.1 (Figure 6D).

**Figure 7:**
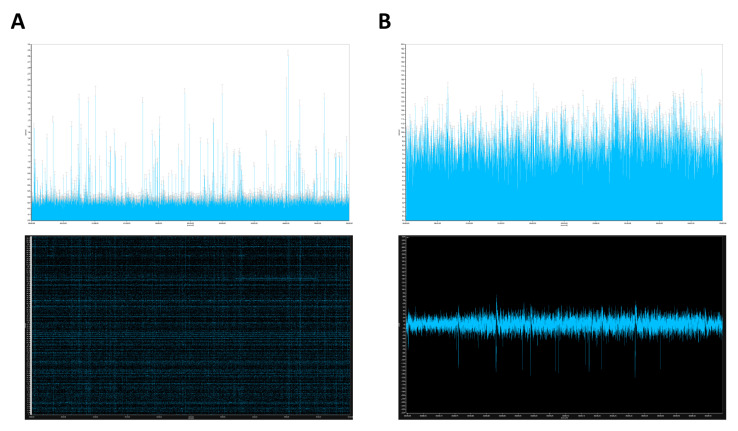
Basal spontaneous networked activity and presence of a subpopulation of strongly active hiPSC-derived MNs in 8-weeks mature cultures. (**A**) AFR of 4096 channels over 5 min of recording (top panel) and related raster plot (bottom panel). (**B**) AFR of the 64 most active channels out of 4096 (top panel) and a representative trace of a single channel showing multiple spikes within 1 s of recording (bottom panel).

To evaluate the network activity in fully mature MNs cultures, we exploited a CMOS-based HD-MEA analysis that has been recently optimized to evaluate this parameter on both primary and hiPSC-derived neurons [38,39]. Spontaneous spike activity was detected with the average firing rate (AFR) analysis in the 8-weeks mature MNs cultures (Figure 7A, top panel). The associated raster plot visibly showed a networked activity of the recorded channels during spiking (Figure 7A, bottom panel). Interestingly, when limiting the AFR to the 64 most active channels, the minimum value resulted to be consistently higher than the 4096 channels AFR maximum value, thus indicating the presence of a highly active subpopulation of MNs (Figure 7B, top panel).

Taken together, the neuronal spontaneous activity results showed an improved basal activity of the culture along with a strongly active subpopulation of MNs following 8 weeks of neuronal maturation. Particularly, the glutamate receptors activity over time clearly denoted the passage from a less mature to a fully mature population of MNs. Interestingly, by increasing the cell density we could detect a high and persistent response following the receptors stimulation also in the 4-weeks mature cultures, which in this condition resulted to be predominantly enriched with MNs showing fully responsive receptors to glutamate agonists (data not shown). However, the higher cell density was detrimental for the long-term cell cultures (data not shown). Another characteristic reported to alter the neuronal differentiation process is the presence of astrocytes in culture, which is known to increase the MNs maturation and spontaneous spike frequency [40]. We therefore speculate that the late-stage spontaneous appearance of astrocytes following 4 weeks of maturation, and their reduced number in our cultures, might be a key factor that contributes to the detection of consistent spontaneous activity only by 8 weeks of maturation and not earlier. Nonetheless, our results on spontaneous activity were in accordance with several protocols from the literature where the authors mostly detected only induced APs rather than spontaneous APs in the MNs cultures [41].

### 3.5. Generation of MNPCs and MNs from hiPSCs from a SMA Patient for Disease Modelling

**Figure 8:**
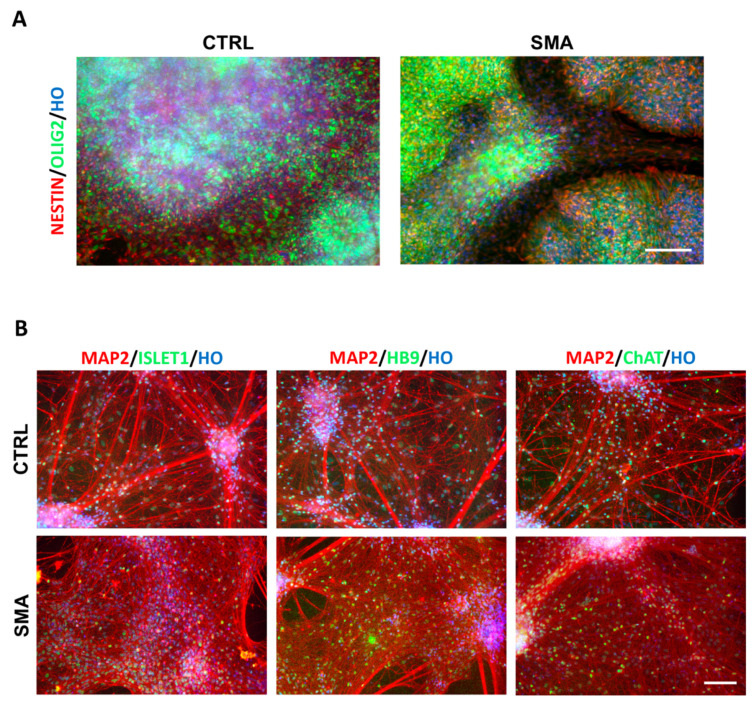
Generation of MNPCs and MNs from SMA patient-derived hiPSCs. (**A**) Representative pictures of DIV13 cultures differentiated from hiPSCs from a healthy individual (CTRL) and an SMA patient. Nuclei are stained with Hoechst (HO). (**B**) Representative pictures of 4-weeks mature MNs differentiated from hiPSCs derived from a healthy individual (CTRL) and an SMA patient. Immunofluorescent analyses for MAP2 along with different MNs markers are shown. Nuclei are stained with Hoechst (HO). Scale bar: 100 µm.

Patient-specific MNs represent valuable in vitro model systems to decipher the pathogenesis of a variety of movement diseases. We thus tested our differentiation procedure to generate MNPCs and mature MNs, starting from an SMA patient-derived and a related (healthy carrier) control hiPSC line. Both the populations rapidly generated neuroepithelial cells organized in neural rosettes and were efficiently patterned into MNPCs. The immunofluorescence analysis on the DIV13 cultures indicated that the cells in culture homogenously co-expressed NESTIN and OLIG2 (Figure 8A).

The expanded MNPCs showed a strongly reduced immunoreactivity to NKX2.2 while retaining OLIG2 immunopositivity, thus confirming their segregation into progenitors restricted to MN lineage (not shown). Following 4 weeks of exposure to the neuronal maturation conditions, the cultures were predominantly populated by MAP2^+ve^ neurons co-expressing HB9, ISLET-1 and ChAT MN markers (Figure 8B).

These results confirmed that the differentiation procedure is applicable to different hiPSC lines to generate nearly pure populations of expandable MNPCs to be further robustly differentiated towards large numbers of MNs to be exploited for in vitro disease modelling approaches.

## 4. Conclusions

We have reported an optimized procedure for generating a highly enriched population of MNPCs in 11–13 days in fully defined monolayer conditions and with a reduced use of small molecules. We showed that these MNPCs can be expanded for at least three passages in culture, allowing the production of large lots of cryopreservable cells retaining their original competence to efficiently generate fully mature functional MNs in culture. Besides the purity and yield of hiPSC-derived MNs, an important challenge in modeling neurological diseases is how to culture these cells physiologically, allowing for long-term cultures. We showed that in our conditions, the MNs cultures can be long-term maintained (we reached a 3-month period; not shown), thus providing the means to study the molecular processes guiding progressive functional maturation, and to analyze the degenerative phenomena associated with the pathologies involving MNs and their functional, networked activity. To this end, we showed for the first time the increasing glutamate receptors functional activity during human MNs maturation, opening to new opportunities for studying the pharmacological excitability over time on these cell populations.

Our results further confirm the important role of the time and range of exposure to key signals during progenitor cells specification to achieve the best efficiency for cell identity maintenance and subsequent neuronal differentiation. The use of fully defined media and the reduced complexity of the differentiation strategy that reduces the number of factors needed for neuralization and patterning, exhibiting a completely xeno-free environment during the whole process, provides a fast and accessible method to be used in human motor neuron disease modelling studies. Given these features, our protocol offers a valid alternative that may be adapted in GMP conditions for translational and clinical purposes and in applications requiring large amounts of nearly homogeneous well-defined target cells, such as for high-throughput screening.

Future efforts will be devoted to generating spinal cord astrocytes to better reproduce a completely controlled environment that leads to MNs maturation and to study glial cells implication and contribution in healthy and pathological conditions, providing additional means to unravel the pathophysiological aspects not yet fully understood.

## Data Availability

The data presented in this study are available in article.

## Supplementary Materials

The following are available online at https://www.mdpi.com/article/10.3390/cells10051127/s1, Detailed Differentiation Protocol; Supplementary Figures Legend; Figure S1: hiPSCs induction into MNPCs in monolayer in patterning condition #2, Figure S2: patterning condition #1 is more efficient in preserving OLIG2 expression along with NKX2.2 loss in hiPSC-derived MNPCs, Figure S3: proliferation potential of MNPCs expanded in CHIR/VPA medium is not significantly affected compared to CTRL conditions, Figure S4: MNPCs differentiate towards neuronal lineage, Figure S5: cultures exposed to patterning condition #1 are more efficient in generating MNs compared to condition #2, Figure S6: intrinsic membrane properties comparison of two maturation stages; Supplementary Video legend; Video S1: representative movie showing MNs’ fast response upon kainate stimulation on cultures exposed for 8 weeks to neuronal maturation conditions.
