# Supplementary material for: A Monolayer System for the Efficient Generation of Motor Neuron Progenitors and Functional Motor Neurons from Human Pluripotent Stem Cells
Source: Cells. 2021 May 7;10(5):1127. doi: 10.3390/cells10051127 (PMC8151197; doi:10.3390/cells10051127)
Supplement: Supplementary file 1 [file cells-10-01127-s001.zip › cells-1186480-supplementary revised/Supplementary data CUTARELLI revised.pdf]

Supplementary data

Detailed protocol for the derivation of MN progenitor cells and functional motor neurons from hPSCs

Neural induction, MNPCs specification and expansion

1. **DIV0:** 70-80% confluent hPSCs cultured in a well of a 6-MW plate are dissociated with EDTA/PBS Dissociation Solution, collected in a centrifuge tube, and spun at 200 rcf for 5 minutes. The supernatant is discarded, the cells resuspend in **Neural Induction Medium (NIM)** and plated at a 1:4-1:6 ratio on a Vitronectin-coated well of a 6-MW plate in NIM supplemented with 10µM Y-27632. Leave 48h at 37°C. Medium is renewed every other day (do not include Y-27632).

2. **DIV6:** Medium is shifted to **NIM** supplemented with RA (100nM) to start the patterning process.

3. **DIV8:** Neural rosettes are gently selected and detached with EDTA/PBS Dissociation Solution, the cells collected in a centrifuge tube and spun at 200 rcf for 5 minutes. The supernatant is discarded, the cells resuspended and plated at a 1:1-1:2 ratio on a Vitronectin-coated well of a 6-MW plate in MNP Patterning Medium (**MNP-PM**) supplemented with 10µM Y-27632. Medium (W/O Y-27632) is renewed every other day.

4. **DIV13:** Cultures at this stage should contain at least 80% of NESTIN^{+ve}/OLIG2^{+ve} MNPCs. Cells are detached by using StemPro Accutase (3-5 minutes at 37°C) and splitted at a1:2-1:3 ratio on Vitronectin-coated 6-MW plate in **MNP Expansion Medium (MNP-EM)** supplemented with 10µM Y-27632. Medium (without Y-27632) is renewed every other day.

5. **DIV15:** Cells can be expanded, as detailed at point 4, for at least three passages (1:2-1:3 split ratio) and/or cryopreserved at this stage.

MNs maturation

6. **DIV0:** To induce motor neurons maturation, MNPCs are dissociated with StemPro Accutase, collected in a centrifuge tube, and spun at 200 rcf for 5 minutes. The supernatant is discarded, the cells resuspended in the desired volume of **MN Maturation Medium (MN-MM)** and 2-2.5 x 10^4 cm^{-1} cells are plated on Vitronectin-coated wells of a 24-MW plate in supplemented with 10µM Y-27632.

7. **DIV2:** The medium is renewed with fresh MN-MM (W/O Y-27632).

8. **DIV4-60:** 50% of the medium is renewed with fresh MN-MM (W/O Y-27632) twice a week to allow neuronal maturation.

Material

EDTA/PBS Dissociation Solution (0.5 mM EDTA in PBS W/O Ca^{2+}/$Mg^{2+)}$
PBS (ThermoFisher Scientific)
StemPro Accutase (ThermoFisher Scientific)
Vitronectin (ThermoFisher Scientific)

Media composition

Neural Induction Medium (NIM)
Essential 6 (ThermoFisher Scientific)
Glutamax, 1% (ThermoFisher Scientific)
Y-27632, 10µM (Santa Cruz Biotechnology)

MNP Patterning Medium (MNP-PM)
Essential 6 (ThermoFisher Scientific)
Glutamax, 1% (ThermoFisher Scientific)
B27, 2% (ThermoFisher Scientific)
RA, 100nM (Focus Biomolecules)
PMN, 2µM (Santa Cruz Biotechnology)
Y-27632, 10µM (Santa Cruz Biotechnology)

MNP Expansion Medium (MNP-EP)
Essential 6 (ThermoFisher Scientific)
Glutamax, 1% (ThermoFisher Scientific)
B27, 2% (ThermoFisher Scientific)
RA, 100 nM (Focus Biomolecules)
PMN, 2 µM (Santa Cruz Biotechnology)
CHIR99021, 3µM (Sigma Aldrich)
VPA, 0.5 mM (Santa Cruz Biotechnology)
Y-27632, 10µM (Santa Cruz Biotechnology)

MN Maturation Medium (MN-MM)
Neurobasal, (ThermoFisher Scientific)
Glutamax, 1% (ThermoFisher Scientific)
B27, 2% (ThermoFisher Scientific)
N2, 1% (ThermoFisher Scientific)
RA, 100nM
PMN, 1.5 µM (Santa Cruz Biotechnology)
GDNF, 10 ng/ml (Peprotech)
BDNF, 10 ng/ml (Peprotech)
IGF-I, 10 ng/ml (Peprotech)
cAMP, 0.2 µM (Santa Cruz Biotechnology)
Y-27632, 10 µM (Santa Cruz Biotechnology)

Supplementary Figures and Video Legend

Figure S1. hiPSCs induction into MNPCs in monolayer in patterning condition #2. Schematic representation of MNPCs and MNs induction, reporting media and factors required for each step.

Figure S2. Patterning condition #1 is more efficient in preserving OLIG2 expression along with NKX2.2 loss in hiPSC-derived MNPCs. The graphs report the number of OLIG2^{+ve} (**A**), OLIG2^{+ve}/NKX2.2^{+ve} (**B**), and OLIG2^{+ve}/NKX2.2^{-ve} (**C**) cells in DIV11 and DIV15 cultures exposed to patterning conditions #1 or #2. The percentage is normalized over the total number of cells assessed by HO staining. Data are expressed as the means ± STDV (n = 3 biologically independent experiments). $p < 0.05$ (*), $p < 0.01$ (**), $p < 0.001$ (***) and $p < 0.0001$ (****) according to Student's *t*-test analysis.

Figure S3. Proliferation potential of MNPCs expanded in CHIR/VPA medium is not significantly affected compared to CTRL conditions. Representative pictures of phospho Histone-H3 (pHH3) immunoreactive cells in MNPCs cultures expanded *in vitro* in CTRL, CHIR and CHIR/VPA conditions for three passages. Scale bar: 100 μm. The graph in the lower panel reports the percentage of pHH3 immunoreactive cells in culture for each medium condition. The percentage is normalized over the total number of cells assessed by HO staining. Data are expressed as the means ± STDV (n = 3 biologically independent experiments). $p < 0.001$ (***), not significant (*ns*) according to ANOVA analysis.

Figure S4. MNPCs differentiate towards neuronal lineage. Graph showing Nestin and β3-Tubulin transcripts levels at different time points during the *in vitro* differentiation procedure. Day 0: hiPSCs; Day 13: MNPCs; Day 20: MNPCs exposed for 7 days to neuronal differentiation conditions; Day 30: MNPCs exposed for 17 days to neuronal differentiation conditions. Data are expressed as the means ± STDV (n = 3 biologically independent experiments).

Figure S5. Cultures exposed to patterning condition #1 are more efficient in generating MNs compared to condition #2. **(A)** Number of TuJ1^{+ve}, MAP2^{+ve} and TuJ1/MAP2 double immunoreactive cells obtained from MNPCs generated in conditions #1 or #2 and exposed for 4 weeks to neuronal maturation medium. The percentage is normalized over the total number of cells assessed by HO staining. **(B)** Graph showing Islet-1, HB9 and ChAT transcripts levels in cultures of MNPCs generated in conditions #1 or #2 and exposed for 4 weeks to neuronal maturation medium. Data are expressed as the means ± STDV (n = 3 biologically independent experiments). $p < 0.01$ (**), $p < 0.001$ (***), not significant (*ns*) according to Student's *t*-test analysis.

Figure S6. Intrinsic membrane properties comparison of two maturation stages. **(A)** Current-Voltage relationship of the representative traces in Fig. 5A, E. The magnitude of inward current was calculated from the maximum amplitude of peak current, while the outward current was calculated from the steady-state current at the end of the pulse. **(B)** Passive membrane properties of 4- and 8-weeks maturation stages calculated from the injection of hyperpolarizing current (0 to -150 pA, 30 pA steps). **(C)** AP waveform measurements of the signals showed in Fig. 5. *$p < 0.05$, according to Student's *t*-test analysis. Each independent sample is plotted as an individual dot.

Video S1. Representative movie showing MNs fast response upon Kainate stimulation on cultures exposed for 8 weeks to neuronal maturation conditions. Cells in resting condition are in green colour. Following agonist stimulation, the occurrence of different degree of red colour indicates different intracellular Calcium concentrations. Noticeably, few red cells already show higher $[Ca^{2+}]i$ before stimulation; on the other hand, non-neuronal cells do not show any intracellular Calcium increase following stimulation.
