# Supplementary figures and images for: A Monolayer System for the Efficient Generation of Motor Neuron Progenitors and Functional Motor Neurons from Human Pluripotent Stem Cells
Source: Cells. 2021 May 7;10(5):1127. doi: 10.3390/cells10051127 (PMC8151197; doi:10.3390/cells10051127)

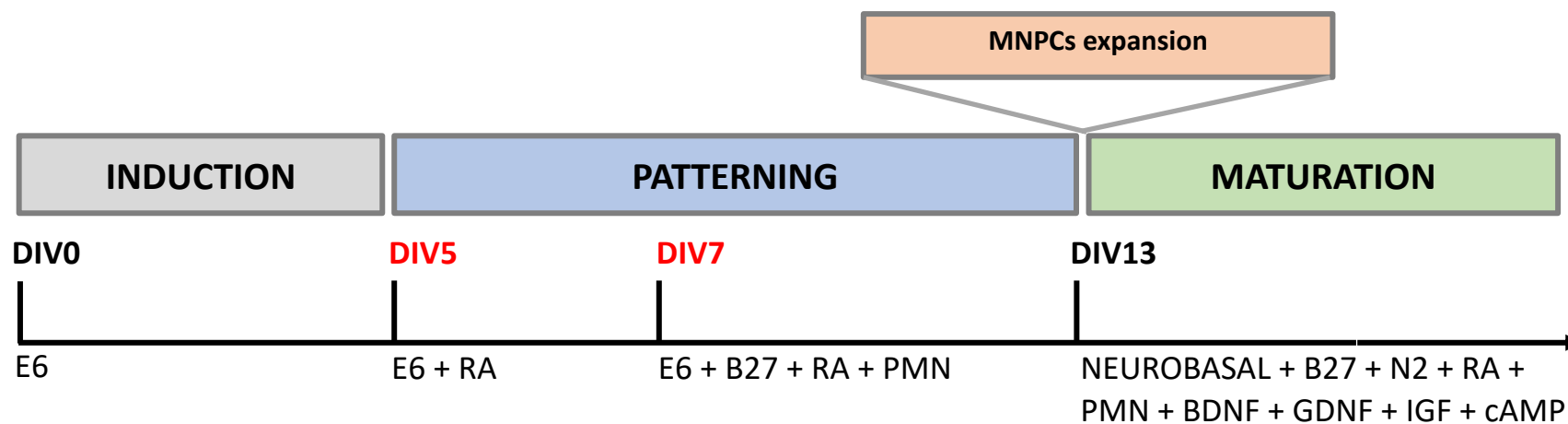

Figure S1.

**A**

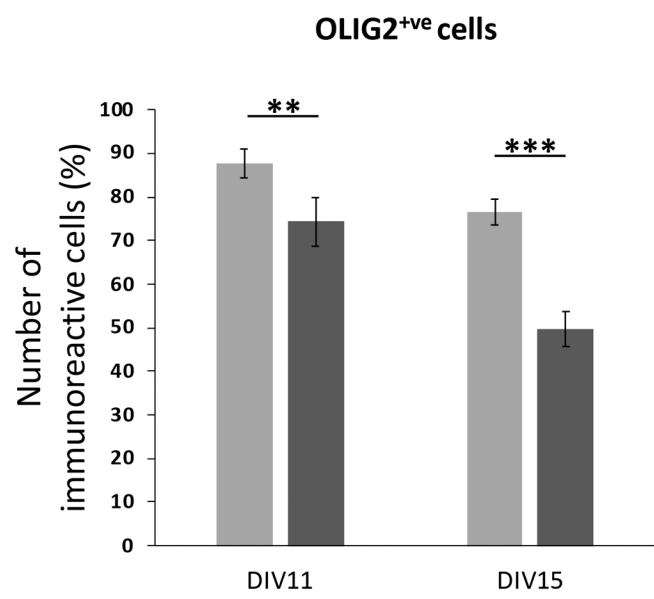

**B**

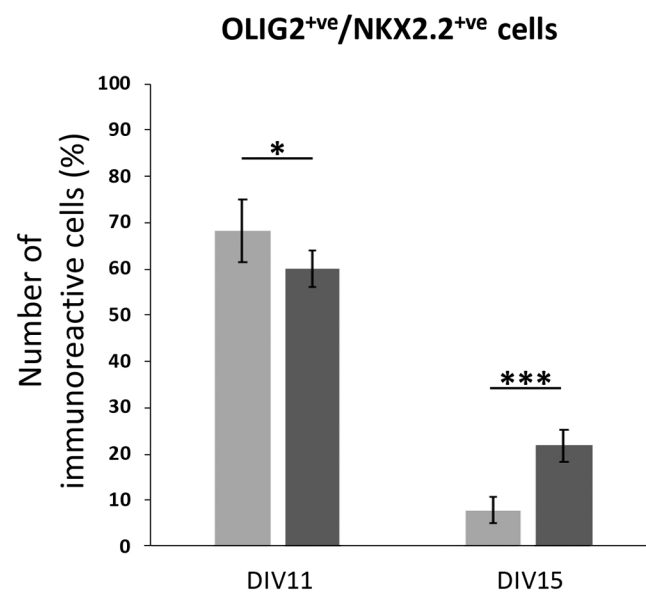

**C**

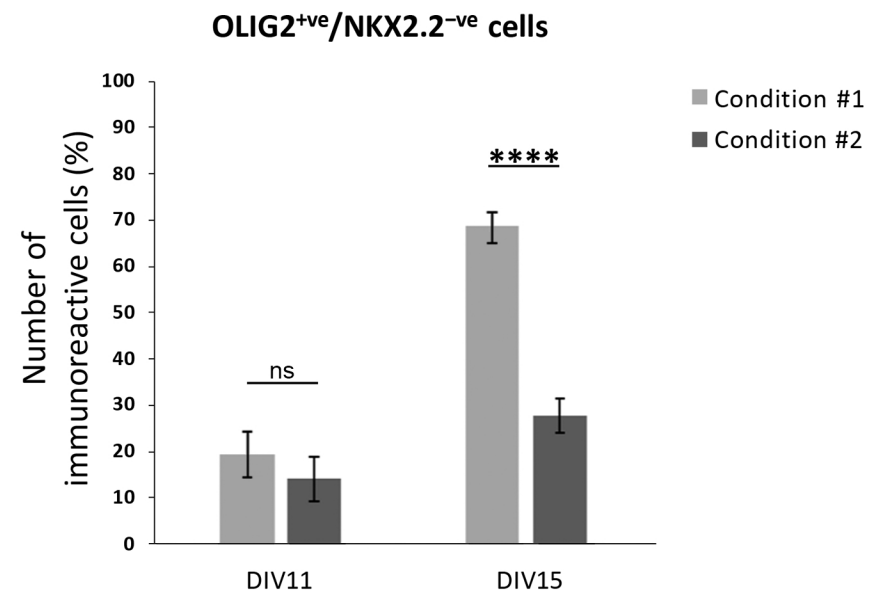

**Figure S2.**

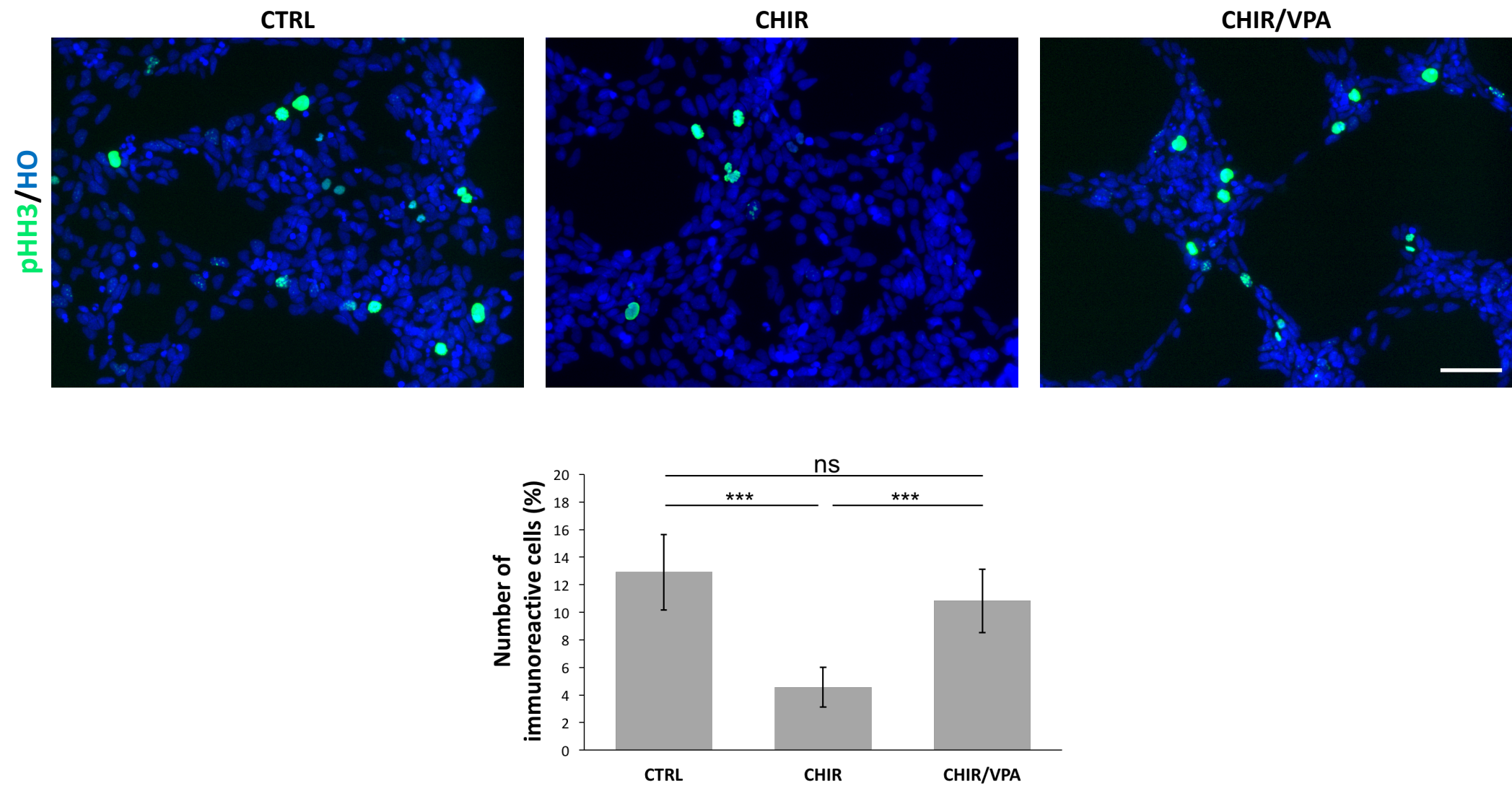

Figure S3.

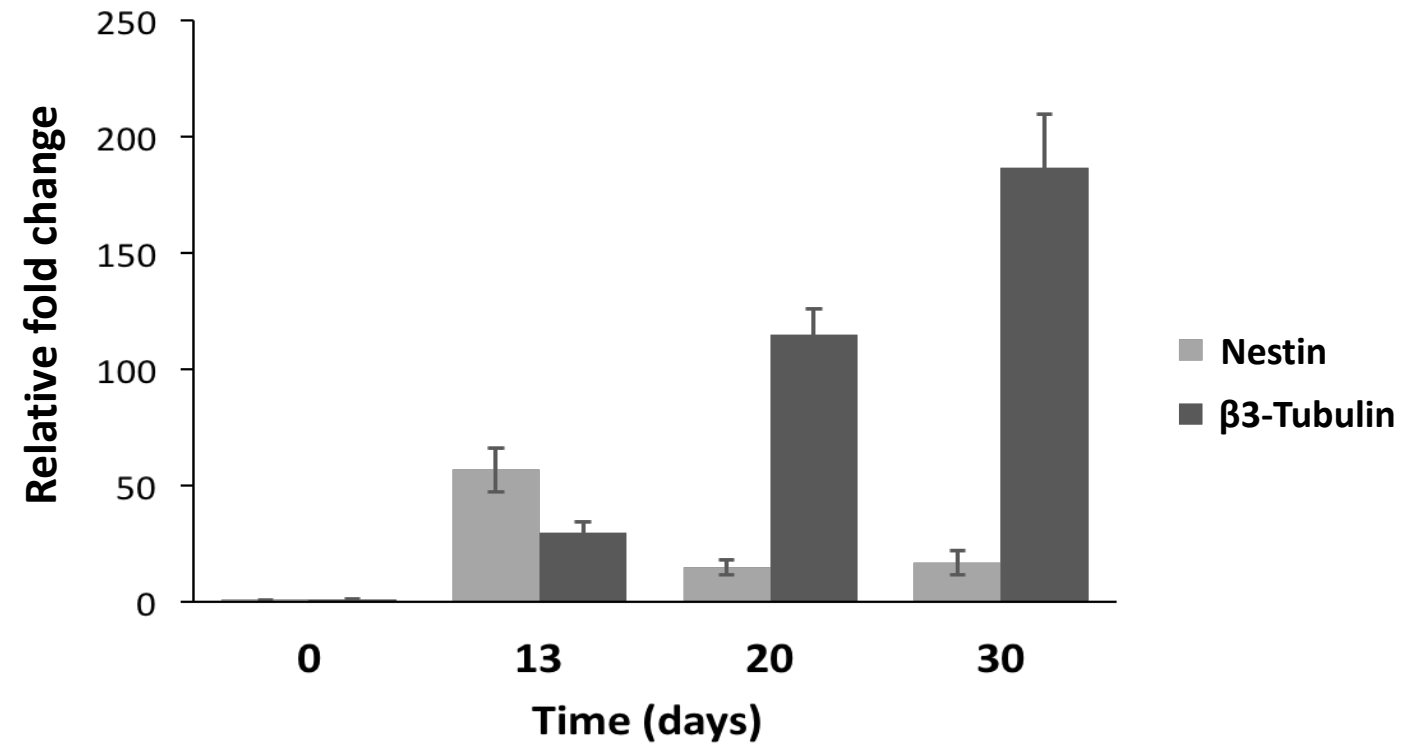

Figure S4.

**A**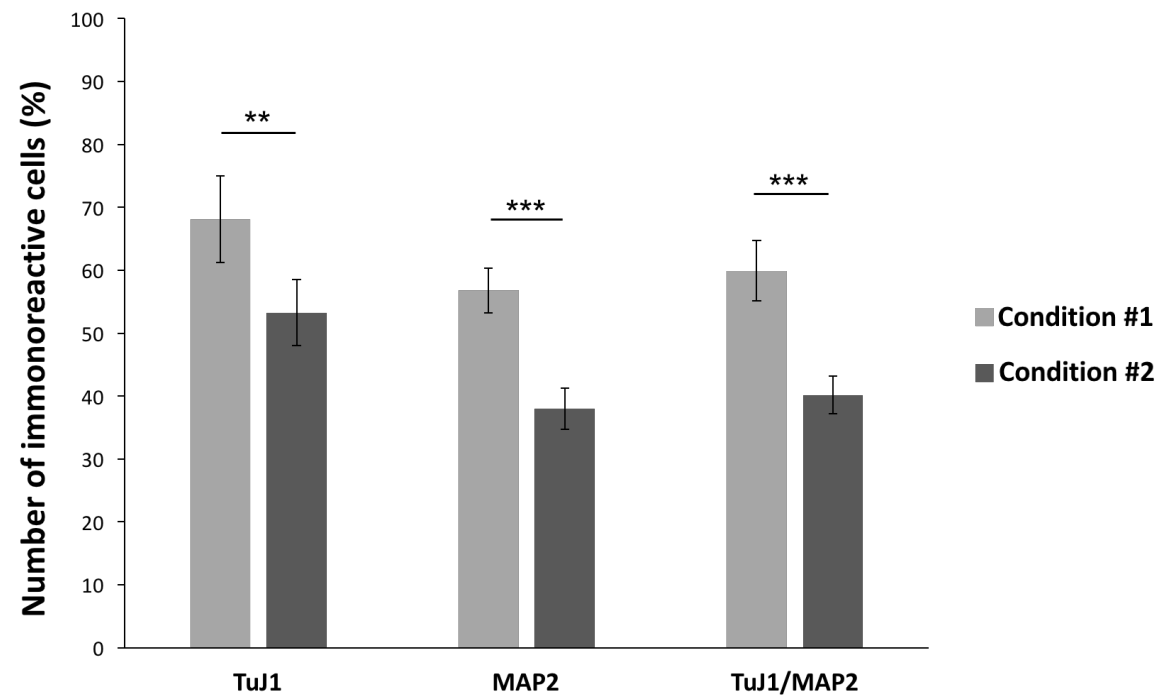**B**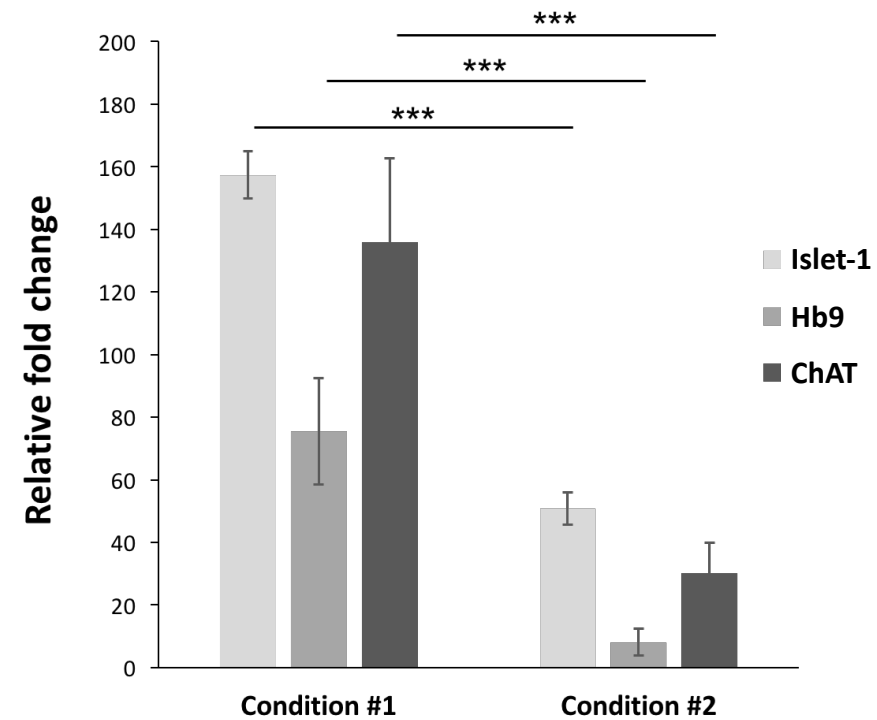

Figure S5.

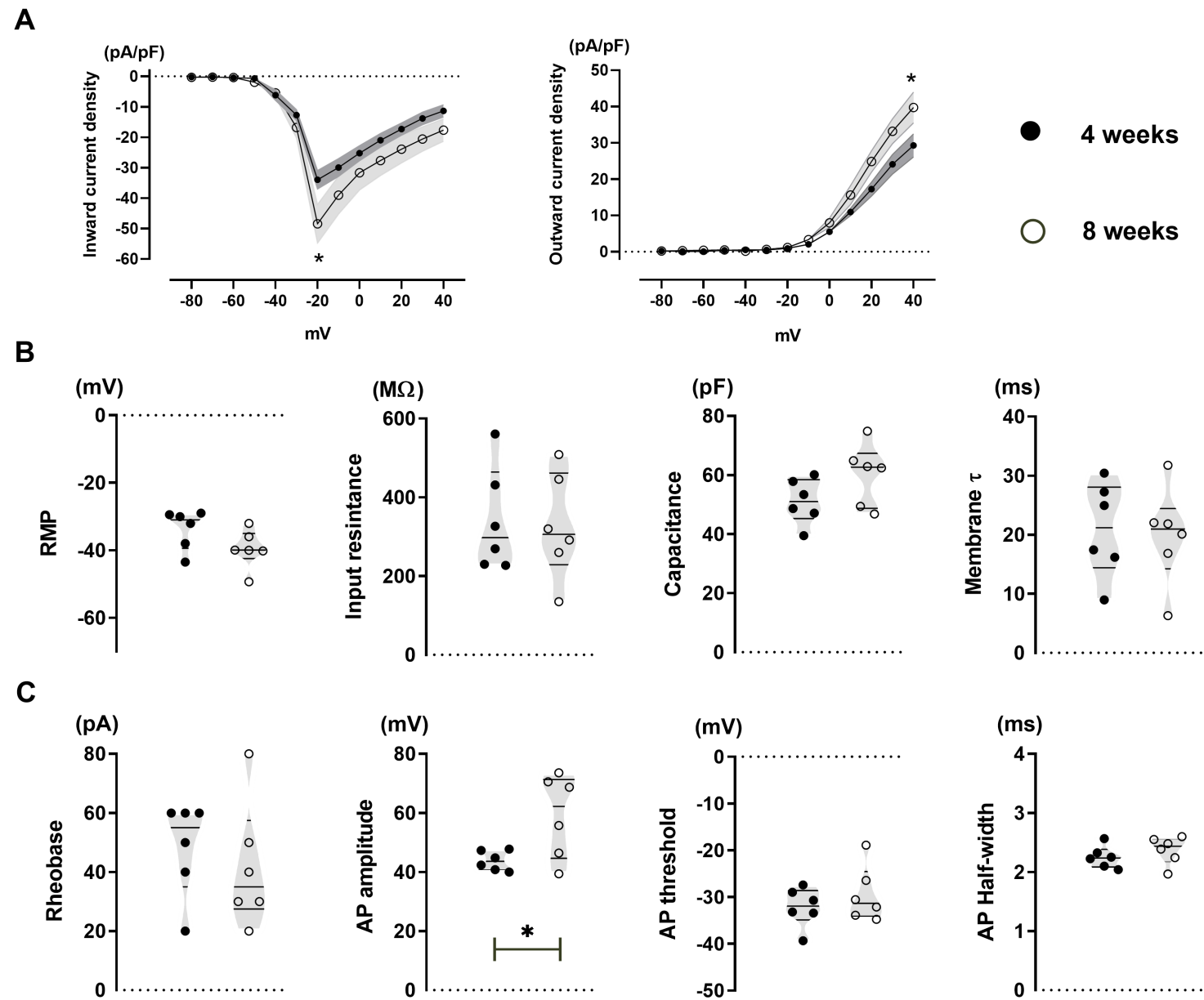

Figure S6.

Supplement: Supplementary file 1 [file cells-10-01127-s001.zip › cells-1186480-supplementary revised/CUTARELLI Supplementary Figures revised.pdf]
